# A Para-Canalicular Abscess Resembling an Inflamed Chalazion

**DOI:** 10.1155/2013/618367

**Published:** 2013-05-27

**Authors:** Diamantis Almaliotis, Elias Nakos, Thomas Siempis, Triantafyllia Koletsa, Ioannis Kostopoulos, Maria Chatzipantazi, Vasileios Karampatakis

**Affiliations:** ^1^Laboratory of Experimental Opthalmology, Medical School, Aristotle University of Thessaloniki, University Campus, 54124 Thessaloniki, Greece; ^2^Laboratory of Anatomical Pathology, Medical School, Aristotle University of Thessaloniki, University Campus, 54124 Thessaloniki, Greece

## Abstract

*Background.* Lacrimal infections by *Actinomyces* are rare and commonly misdiagnosed for long periods of time. They account for 2% of all lacrimal diseases. *Case Report.* We report a case of a 70-year-old female patient suffering from a para-canalicular abscess in the medial canthus of the left eye, beside the lower punctum lacrimale, resembling a chalazion. Purulence exited from the punctum lacrimale due to inflammation of the inferior canaliculus (canaliculitis). When pressure was applied to the mass, a second exit of purulence was also observed under the palpebral conjunctiva below the lacrimal caruncle. A surgical excision was performed followed by administration of local antibiotic therapy. The histopathological examination of the extracted mass revealed the existence of actinomycosis. *Conclusion.* Persistent or recurrent infections and lumps of the eyelids should be thoroughly investigated. *Actinomyces* as a causative agent should be considered. Differential diagnosis is broad and should include canaliculitis, chalazion, and multiple types of neoplasias. For this reason, in nonconclusive cases, a histopathological examination should be performed.

## 1. Introduction

The word actinomycosis means “ray fungus.” These organisms may resemble fungi because of their filamentous semblance. *Actinomycetes* are a group of anaerobic, Gram-positive bacteria that vary phylogenetically, but are morphologically alike. The genus *Actinomyces* consists of a group of 42 species and 2 subspecies. The most common agent, concerning human diseases, among the *Actinomyces* species is *A. israelii.* However, there are less frequent species such as *A. naeslundii*, *A. odontolyticus*, *A. viscosus*, *A. meyeri*, and *A. gerencseriae*. Recently, new species were identified such as *A. europaeus*, *A. neuii*, *A. radingae*, *A. graveenitzii*, *A. turicensis*, *A. cardiffensis*, *A. houstonesis*, *A. hongkongensis,* and *A. funkei*. 

An infection due to *Actinomyces* may be related to damage of the normal physical barriers, such as the mucosal membranes in the mouth or the gastrointestinal tract. These infections can result in abscess formation and/or chronic progression [1].

*Actinomyces* may cause thoracic, abdominal, pelvic, central nervous system, musculoskeletal, or soft tissue infections. Ocular infections by *Actinomyces* are uncommon.

We present a rare case of an actinomycosis beside the lower punctum lacrimale, resembling a recurrent chalazion.

## 2. Case Report

A 70-year-old woman was referred to our clinic because of a tender, red, and painful swelling accompanied by purulent secretions in the left medial canthus, subadjacent to the lower punctum lacrimale. According to her ocular history, this condition was first noticed 2 years ago as mild tenderness in the aforementioned area followed by purulent discharge. The patient underwent treatment with steroid and antibiotic drops at various time intervals. The diagnosis was not confirmed for a long time, and the case was characterized as a recurrent chalazion. 

Upon clinical examination, an inflamed swelling was observed, and the patient reported slight pain. Ocular examination revealed normal visual acuity. The swelling was firm and nodular like a chalazion. The punctum was found to be pouting with expression of pus upon pressure over the swelling. This fact confirmed the existence of canaliculitis. A second exit of purulence was observed through the palpebral conjunctiva below the lacrimal caruncle.

**Figure 1:**
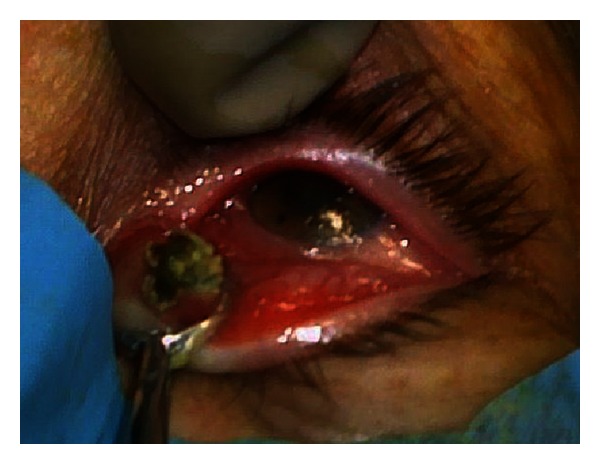
The incision perpendicularly revealed the content of the abscess.

The chosen treatment included the “chalazion” surgery technique. An incision was performed through the inner palpebral side, and a grey mass like a fungus was extracted under pressure (Figure 1). The material was sent to a histopathology laboratory for evaluation.

The cavity was drained. Local and systemic antibiotic therapy was administered: coll tobramycin 1 × 4, coll ofloxacin 1 × 4, and tabs ciprofloxacin 500 mg × 2.

After the surgery, the size of swelling progressively decreased (in about 10 days) followed by the disappearance of purulent discharge.

**Figure 2:**
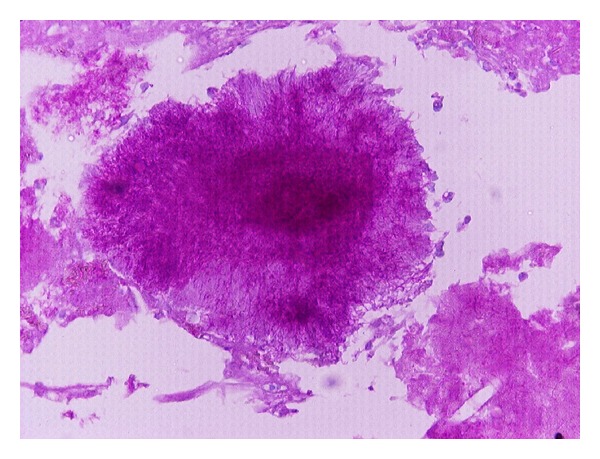
*Actinomyces* colony PAS ×400.

The histopathological diagnosis revealed the existence of actinomycosis (Figure 2).

## 3. Discussion

It is well known that *Actinomycetes* are colonizers of the human body. They might be present in the oral cavity, the colon, or the urogenital tract of humans and animals [2]. However, person-to-person transmission has never been documented. The infection due to *Actinomyces* is connected with the breakdown of the normal physical barriers, such as the natural mucosal membranes in the gastrointestinal tract and mouth. Therefore, actinomycosis is often associated with dental extractions and caries, erupting secondary teeth, gingivitis, and gingival trauma. In those cases, infection is caused via direct invasion, because of the break in mucosal integrity. The spread of *Actinomyces* by metastatic or hematogenous routes is uncommon. The progression of the infection is chronic and leads to abscess formation with fistulous tracts and draining sinuses. Invasion and destruction of the surrounding structures are observed, as actinomycosis generally spreads without regard to anatomic barriers [3]. 

Except ocular infection, *Actinomyces* might cause thoracic, abdominal, pelvic, central nervous system, musculoskeletal, or soft tissue infection. Cases of endocarditis, pericarditis, bacteremia, osteomyelitis, and chorioamnionitis have also been published [4].

Actinomycosis is found in all age groups, with higher incident rates in the midlife and lower in the older ages. Depending on studies and case reports, males are more frequently infected in comparison to females. However, our case concerns a 70-year-old woman. Nowadays, incidence of actinomycosis has decreased due to improved dental hygiene and the use of antibiotics. 

In our case, the para-canalicular abscess was caused by actinomycotic canaliculitis.

Apart from canaliculitis *Actinomyces* is documented to cause various ophthalmic infections, such as blepharitis, conjunctivitis, keratitis, dacryocystitis, and postoperative endophthalmitis. Usually in those cases there is no generalized systemic invasion [5].

The first available report of an ocular infection due to a species of *Actinomyces* comes from Roussel et al. [6]. In general, there are not many published cases of ocular infections by *Actinomyces*. 

Some interesting cases are an actinomycotic granule of the caruncle [7], a case of actinomycosis of the orbit described by Sullivan et al. [8], a similar case of lacrimal canaliculitis by Vagarali et al. [9], and a series of seven patients with *Actinomyces* canaliculitis from Briscoe et al. in Israel [10]. In the study of Sullivan, the authors assumed that the infection was a result of direct expansion from an instance of dental caries. History of trauma either a human bite or perforating injury with contamination from outside can also cause actinomycosis.

Canaliculitis is an uncommon chronic condition—usually undiagnosed for long periods of time—that usually arises from the Gram-positive bacteria *Actinomyces israelii* (streptothrix) but may be also due to *Nocardia* fungi (*Candida* and *Aspergillus*) and viruses (HSV, VZV). The clinical features of canaliculitis are the following [9, 10]:

- unilateral epiphora, recurrent nasal conjunctivitis, inflammation of the punctum and the canaliculus, expression of discharge, or concretions from the canaliculi,
- in *Actinomyces* infection there are bright yellow secretions (sulphur granules). The lacrimal sac is not swollen and both sac and nasolacrimal duct are patent.

Our patient had the aforestated symptoms and signs for a long period of time. Diagnosis of canaliculitis is achieved through clinical, microbiological, and histopathological examination in order to exclude neoplasias [10].

In general, the process of infection in actinomycosis can be confused with a *Nocardia* infection. Moreover, in many cases it could not be differentiated from chalazion or neoplasias [2].

Thus, the differential diagnosis of tumors simulating chalazion is broad; mucinous sweat gland adenocarcinoma of the eyelid [11], primary cutaneous adenoid cystic carcinoma [12], schwannoma of the upper eye lid [13], and trichilemmal cyst [14] are some rare cases that were initially misdiagnosed as chalazion.

As far as the treatment of canaliculitis is concerned surgical intervention is required to effect a cure. Canaliculotomy and curettage are the treatment of choice followed by prolonged systemic antibiotic therapy and sulfacetamide eye drops [9, 10]. *Actinomyces* are susceptible to many antibiotics, such as penicillins, cephalosporins, clindamycin, carbapenems, and tetracycline [1].

According to Briscoe et al., instillation of aqueous penicillin or povidone iodine and meticulous repair of the canaliculus are not necessary [10].

Irrigation should also be considered with penicillin G 100,000 u/mL or iodine 1%.

Last but not least, there have been published cases of elimination of some species of *Actinomyces* based on broad-spectrum antibiotics like ciprofloxacin and cefazolin, without the need of invasive procedures, such as canalicular curettage and canaliculotomy. More specifically, canalicular expression followed by topical antibiotic drops and canalicular irrigation with antibiotic solution according to the sensitivity pattern of the microorganism was found to be effective in all cases of an Indian study that included 12 patients with canaliculitis. This treatment has the advantage of eliminating the risk of iatrogenic canalicular scarring with preservation of lacrimal pump function [15].

## 4. Conclusion

Canaliculitis can masquerade at times to a degree that makes it easy to overlook its diagnosis. As a result, any patient presenting with features of epiphora, chronic purulent conjunctivitis, a palpably thickened canaliculus, and yellow punctual discharge on digital expression, exploratory canaliculotomy should be considered to rule out the presence of *Actinomyces*. Finally, persistent or recurrent lumps of the eyelids are suspicious and should be thoroughly investigated. Differential diagnosis is broad and should include abscesses caused by canaliculitis, chalazion and multiple types of neoplasias. For this reason, in non-conclusive cases, a histopathological examination should be performed.
